# Functional divergence of the pigmentation gene melanocortin-1 receptor (*MC1R*) in six endemic *Macaca* species on Sulawesi Island

**DOI:** 10.1038/s41598-022-11681-z

**Published:** 2022-05-09

**Authors:** Xiaochan Yan, Yohey Terai, Kanthi Arum Widayati, Akihiro Itoigawa, Laurentia Henrieta Permita Sari Purba, Fahri Fahri, Bambang Suryobroto, Hiroo Imai

**Affiliations:** 1 Molecular Biology Section, Center for the Evolutionary Origins of Human Behavior, Kyoto University, Inuyama, Japan; 2 Department of Evolutionary Studies of Biosystems, The Graduate University for Advanced Studies, Hayama, Japan; 3 Department of Biology, IPB University, Bogor, Indonesia; 4 Department of Biology, Tadulako University, Palu, Indonesia

**Keywords:** Proteins, Molecular evolution

## Abstract

Coat color is often highly variable within and between animal taxa. Among hundreds of pigmentation-related genes, melanocortin-1 receptor (*MC1R*) plays key roles in regulating the synthesis of the dark eumelanin and the red–yellow pheomelanin. The six species of macaques that inhabit Sulawesi Island diverged rapidly from their common ancestor, *M. nemestrina.* Unlike most macaques, Sulawesi macaques commonly have a dark coat color, with divergence in shade and color pattern. To clarify the genetic and evolutionary basis for coat color in Sulawesi macaques, we investigated the *MC1R* sequences and functional properties, including basal cAMP production and α-MSH-induced activity in vitro. We found fixed non-synonymous substitutions in *MC1R* in each species. Furthermore, we found that six species-specific variants corresponded with variation in agonist-induced and basal activity of MC1R. Inconsistent with the dark coat color, four substitutions independently caused decreases in the basal activity of MC1R in *M. hecki, M. nigra, M. tonkeana,* and *M. ochreata*. Selective analysis suggested *MC1R* of *M. nigra* and *M. nigrescens* underwent purifying selection. Overall, our results suggest that fixed differences in *MC1R* resulted in different functional characteristics and might contribute to divergence in color among the six Sulawesi macaque species.

## Introduction

Animals exhibit extreme coat color variation between and within species. The trait is often a target of selection because even small changes in coat color can have significant implications for camouflage, communication, and thermoregulation. More than 100 genes are related to coat color determination^1^. In vertebrates, melanocortin-1 receptor (*MC1R*) is among the most well-studied and important coat color genes, with a key role in switching between brown-to-black eumelanin and yellow-to-red pheomelanin.

The *MC1R* gene encodes a G-protein coupled receptor (GPCR), consisting of an N-terminal domain, seven hydrophobic transmembrane domains, and a carboxy terminal domain^2^. MC1R is expressed primarily on melanocytes, where it plays a key role in the regulation of melanin pigmentation^3^. It is activated by the agonist α-melanocyte-stimulating hormone (α-MSH), thereby increasing intracellular cAMP levels via the activation of adenylyl cyclase. As a consequence, there is a switch in production from pheomelanin to eumelanin in melanocytes^4^. In contrast, the antagonist agouti-signaling protein (ASIP) depresses MC1R activation and promotes phaeomelanin production.

The role of *MC1R* mutations in coat pigmentation has been studied extensively in domestic and laboratory mammals and birds^5^. MC1R variants have been functionally characterized in several domestic species and, more recently, in beach mice^6^ and mammoths^7^. Several gain-of-function MC1R mutations result in increased eumelanin production in coats (e.g., in chicken, pig, and sheep^8–10^), whereas loss-of-function mutations result in increased pheomelanin production in skin and cause red hair in humans^11,12^ and white lizards at White Sands^13^. However, most studies have concentrated on the effects of MC1R within species, and little is known about divergence in MC1R function between closely related species.

**Figure 1:**
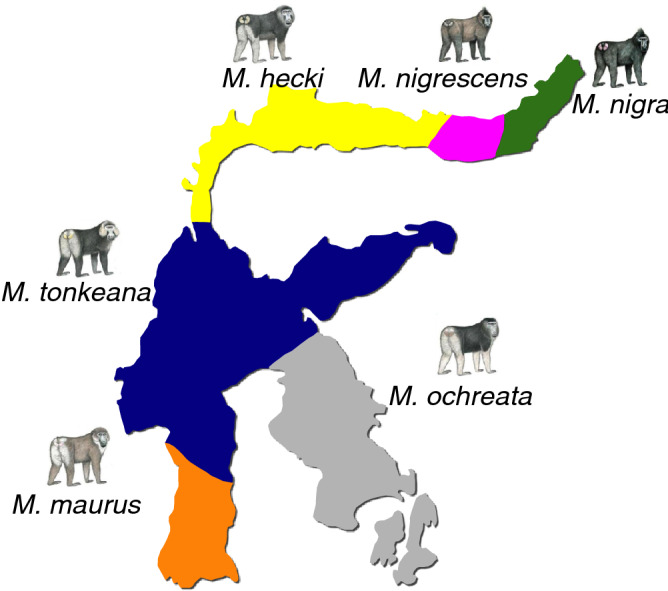
Distribution of six allopatric *Macaca* species on Sulawesi island.

Haitina et al. found that in contrast to humans, lemurs, and platyrrhines, MC1R of catarrhines displays strong functional conservation, including dose-dependent α-MSH binding and high basal activity^14^. In particular, MC1R of *M. nemestrina* shows dose-dependent α-MSH binding and the highest basal activity in the 9 phylogenetically diverse primate species^14^. In the genus *Macaca*, the functional diversity of MC1R is of interest because light–dark coat color variation is a prominent type of phenotypic diversity across species and populations. However, it is not clear whether the functional properties of MC1R are conserved in other *Macaca* species with prominent coat color variation. Nakayama et al. further detected 28 amino acid substitutions among 18 macaque species. They suggested that 7 amino acid substitutions at evolutionarily conserved sites might influence the function of MC1R in macaques distributed on Sulawesi Island (Sulawesi macaques)^15^. Notably, distinguished from most macaques, Sulawesi macaques display a conspicuous dark coat color, with variation in darkness and color pattern among species^16^. Generally, *M. nigra, M. nigrescens*, *M. tonkeana* and *M. maurus* are the species which the color variation is slight among the body parts. In these four species, *M. maurus* has a lighter color in all body parts. *M. tonkeana* has clearly defined pale brownish-gray to pale ochraceous-buff in cheek whisker. *M. nigra* is very dark in all body parts. *M. nigrescens* is lighter than *M. nigra* in the back. On the other hand, *M. hecki* and *M. ochreata* display wide variation of coat color in body parts. Both *M. hecki* and *M. ochreata* are very light in hind-shanks. *M. hecki* is slight light in the forearm, thigh, and cheek whisker. The back of *M. ochreata* is very dark, nearly as dark as *M. nigra*, but the forearm is very light^16^. In a relatively short period of time, Sulawesi macaques have diversified exponentially into six morphologically distinct species from a common ancestor with *M. nemestrina*^17^. The six species are distributed in allopatry on Sulawesi Island (Fig. 1). The evolutionary relationships among these species have been studied by hemoglobin^18^, RAD-seq^19^ and mitochondrial DNA analyses (mtDNA)^20^. Relative to *M. nemestrina*, an extant ancestor of Sulawesi macaques, darkening of the coat color is thought to have been necessary for the evolution of Sulawesi macaques on Sulawesi Island. Given the lack of predation pressures on Sulawesi macaques, the dark pelage was not likely to function as a form of camouflage; instead, it might be an adaptation to terrestrial living and contribute to thermoregulation^16^. However, the evolutionary processes and genetic basis of divergence in color are still unclear. In this study, to clarify how color variation was generated and maintained in Sulawesi macaques, we investigated *MC1R* diversity and its functional characteristics in vitro. We sequenced *MC1R* of about 10 individuals of each Sulawesi macaque species to evaluate sequence diversity. In addition, we pharmacologically characterized MC1R to examine the influence of genetic variation on biochemical function.

## Results

### Genotyping and selection analysis

**Figure 2:**
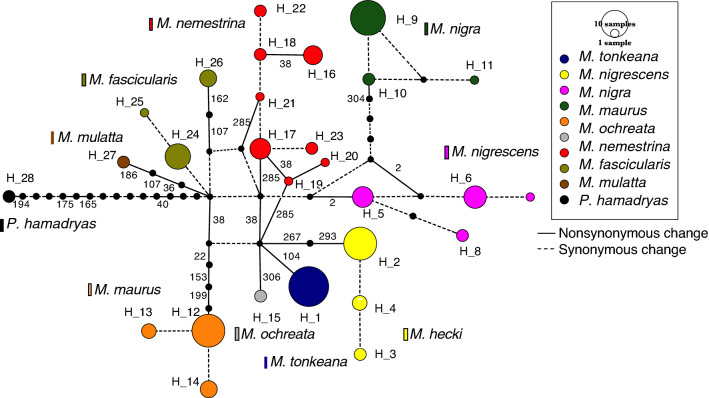
Haplotype network for *MC1R* in Sulawesi macaques, *M. nemestrina*, *M. fascularis*, and *M. mulattta. P. hamadryas* was used as the outgroup. Each circle represents a distinct nucleotide haplotype. The size of a circle is proportional to the allele frequency. Each color represents a different species. Every mutation (nucleotide substitution) is shown as a 1-step edge. Line styles indicate the mutation type. Positions of non-synonymous substituions are indicated on the network branches.

We determined the *MC1R* nucleotide sequences to investigate polymorphisms in six endemic *Macaca* species on Sulawesi Island. From 51 individuals, we identified 15 alleles based on combination of 26 single-nucleotide polymorphisms (SNPs) (Supplementary Table S1), including 10 previously reported nonsynonymous SNPs^15^ (Table 1). In particular, each species exhibited distinct fixed amino acids. All 10 nonsynonymous substitutions (P2R, P22L, M38V, G104S, H153P, M199L, C267Y, I293V, E304G, and R306C) were responsible for six species-specific haplotypes. Sulawesi macaques were separated into two clusters, one is the northern cluster, including *M. nigra* and *M. nigrescens*, the other one is the southern cluster, including *M. hecki, M. tonkeana, M. maurus* and *M. ochreata* (Fig. 2). Ancestral sequences of northern and southern cluster were constructed. Altogether, 9 of the 10 amino acid differences were species-specific, and one amino acid residue (site 38) distinguished *M. nigra* and *M. nigrescens* (northern cluster, 38 V) from the other four species (southern cluster, 38 M).

**Table 1:** Eleven non-synonymous substitutions in Sulwesi macaques and *M. nemestrina*.

MC1R(317AA)	N		TM1	EL1	IL2	TM5	EL3	TM7		C	
	2	22	38	104	153	199	267	285	293	304	306
Consensus N	P	P	V	G	H	M	C	A	I	E	R
Consensus S			M								
*M. nemestrina A*								T			
*M. nemestrina B*			M					T			
*M. nigra*										G	
*M. nigrescens*	R										
*M. hecki*			M				Y		V		
*M. tonkeana*			M	S							
*M. maurus*		L	M		P	L					
*M. ochreata*			M								C

Ten non-synonymous substitutions were found in Sulawesi macaques and one non-synonymous substitution (A285T) was found in *M. nemestrina.* All substitutions were specific to a single species, except at amino acid residue 38, which showed polymorphism in *M. nemestrina* and shared variation between species (38V, shared by *M. nigra* and *M. nigrescens*; 38M, shared by *M. hecki. M. tonkeana, M. maurus,* and *M. ochreata*).

*N* N-terminal, *TM* transmembrane domains, *IL* intracellular loop, *EL* extracellular loop, *C* C-terminal corresponds to the different MC1R domains.

**Figure 3:**
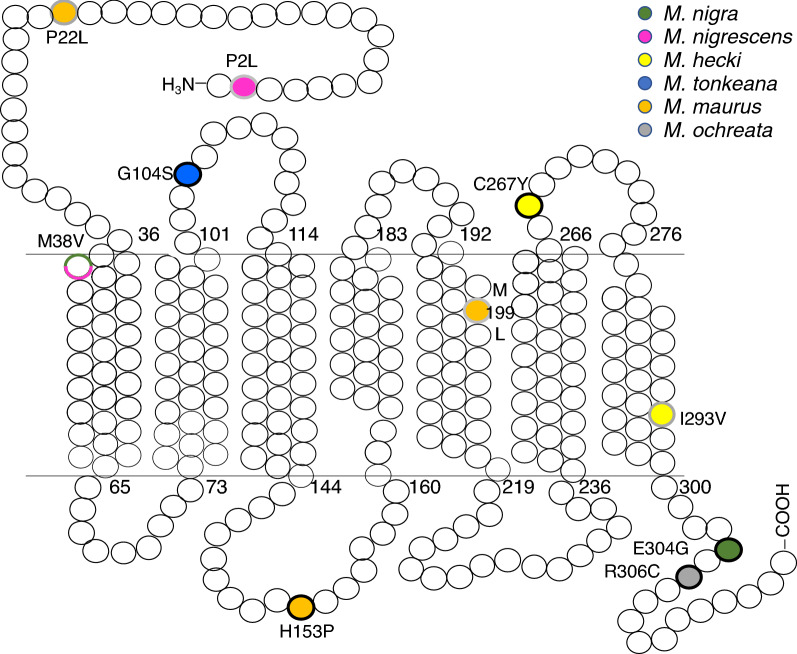
Schematic transmembrane topology for MC1R of Sulawesi macaques. Ten non-synonymous substitutions are shown. Each color represents a different species. The substitutions outlined in black are deleterious, as predicted by both SIFT and PROVEAN analyses.

Each species showed specific nonsynonymous substitutions in a different part of MC1R. In *M. hecki*, all individuals shared two specific substitutions, C267Y and I293V, located in the third extracellular loop (EL3) and the seventh transmembrane region (TM7) of the receptor, respectively (Fig. 3). In *M. nigra,* the specific E304G substitution resulted in a change from a negatively charged residue to an uncharged residue in the C terminal domain of the receptor. In *M. nigrescens*, the P2R substitution resulted in a change from a nonpolar to positively charged residue in the N terminal region of the receptor. In *M. tonkeana*, the conservative substitution G104S resulted in a change from a nonpolar to polar residue in EL1. *M. maurus* possessed the most amino acid substitutions, including P22L, H153P, and M199L. SIFT (Sorting Intolerant From Tolerant; ≤ 0.05) and PROVEAN (Protein Variation Effect Analyzer; ≤  − 2.50) analyses consistently showed that five (G104S, H153P, C267Y, E304G, and R306C) of the substitutions occurred at evolutionarily conserved sites and may alter the functional characteristics of the MC1R receptor (Supplementary Table S2), consistent, in part, with previous results^15^.

To detect positive selection in *MC1R* in Sulawesi macaques, we applied three models using the PAML package: a branch model, site model, and branch-site model. First, using a site model to investigate selection throughout the phylogeny based on RADseq data^19^, we did not detect sites under positive selection, details seeing in Supplementary Table S3. We further calculated ω values for the lineages with melanism (*M. nigra* and *M. nigrescens*), the other four Sulawesi macaques, and *M. nemestrina* and *M. mulatta* using a branch model to examine selective constraints on the black coat color lineage. The ω values for the melanism lineage (ω = 0.086) were significantly lower than those for other Sulawesi macaques (ω = 0.968) and the lineage including *M. nemestrina* and *M. mulatta* (ω = 0.856) (Supplementary Fig. S1). These results suggested that species with melanism underwent purifying selection. We further examined positively selected codon sites by a branch-site model. The null model was not rejected, and no positively selected sites were found.

### Constitutive and α-MSH-induced cAMP production of MC1R variants

**Figure 4:**
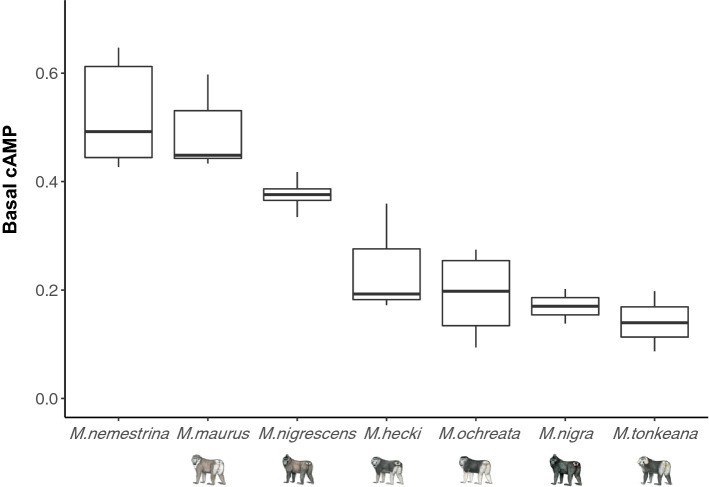
Basal cAMP production of species-specific MC1R variants in Sulawesi macaques. Basal cAMP production was normalized to the maximum cAMP production under activation by 20 µM forskolin. *M. nigra, M. ochreata, M. hecki,* and *M. tonkeana* MC1R showed significantly lower basal cAMP production than levels for *M. nemestrina* and *M. maurus* (Pairwise *t*-test with BH correction, P < 0.05)*. M. nigrescens* displayed intermediate basal cAMP production, significantly lower than that of *M. nemestrina* (Pairwise *t*-test with BH correction, P < 0.05).

The positive control, MC1R of *M. nemestrina*, exhibited high basal activity and a dose-dependent response to the agonist α-MSH, consistent with the results of Haitina et al.^14^. The system was further used to evaluate basal cAMP production and agonist-induced cAMP activity by in vitro cAMP assays for all six species-specific MC1R variants of Sulawesi macaques. Except for *M. maurus* MC1R, basal cAMP levels were markedly lower in cells expressing all variants than in positive control cells expressing *M. nemestrina* MC1R (Fig. 4).

MC1R of *M. maurus* (Δ*F/*Δ*F*_*max*_ = 0.494 ± 0.028) exhibited comparable high basal cAMP production to that in cells expressing *M. nemestrina* MC1R (Δ*F/*Δ*F*_*max*_ = 0.543 ± 0.023). However, MC1R of *M. hecki* (Δ*F/*Δ*F*_*max*_ = 0.241 ± 0.020)*, M. ochreata* (Δ*F/*Δ*F*_*max*_ = 0.204 ± 0.029), *M. tonkeana* (Δ*F/*Δ*F*_*max*_ = 0.140 ± 0.025), and *M. nigra* (Δ*F/*Δ*F*_*max*_ = 0.182 ± 0.025) exhibited low basal cAMP production, with significantly lower levels than those of cells expressing *M. nemestrina* and *M. maurus* MC1R (Table 2). We observed intermediate basal cAMP production in cells expressing *M. nigrescens* MC1R (Δ*F/*Δ*F*_*max*_ = 0.374 ± 0.026). These results showed that MC1R exhibits agonist-independent constitutive signaling in most Sulawesi macaques, which is stronger in *M. nemestrina* and *M. maurus* than in other Sulawesi macaques (pairwise *t*-test, P < 0.05, BH-adjusted; Supplementary Table S4).

**Table 2:** Summary of basal cAMP production and maximun cAMP production.

Variants	Basal cAMP production (ΔF/ΔF_max_)	Maximum cAMP production (ΔF/ΔF_max_)	EC_50_ (nM)
*M. nemestrina*	0.543 ± 0.023	0.937 ± 0.038	0.709 ± 0.363
*M. maurus*	0.494 ± 0.028	0.959 ± 0.048	0.663 ± 0.339
*M. nigrescens*	0.374 ± 0.026	0.876 ± 0.052	0.968 ± 0.490
*M. hecki*	0.241 ± 0.020	ND	ND
*M. ochreata*	0.204 ± 0.029	0.883 ± 0.060	1.125 ± 0.477
*M. nigra*	0.182 ± 0.025	0.597 ± 0.048	1.458 ± 0.803
*M. tonkeana*	0.140 ± 0.025	0.915 ± 0.054	1.886 ± 0.583
Y267C	0.314 ± 0.033	0.891 ± 0.054	0.316 ± 0.180
S104G	0.313 ± 0.030	0.928 ± 0.054	0.696 ± 0.304
P153H	0.310 ± 0.019	0.872 ± 0.036	1.164 ± 0.306
G304E	0.271 ± 0.023	0.849 ± 0.031	0.485 ± 0.145

‘Basal cAMP production’ is the cAMP accumulation without agonist stimulation; ‘Maximum cAMP production’ is the cAMP accumulation which reaches saturation in response to 100 nM α-MSH. The results are presented as mean ± SEM obtained from at least 3 times independent experiments. ND indicates ‘not determined’.

**Figure 5:**
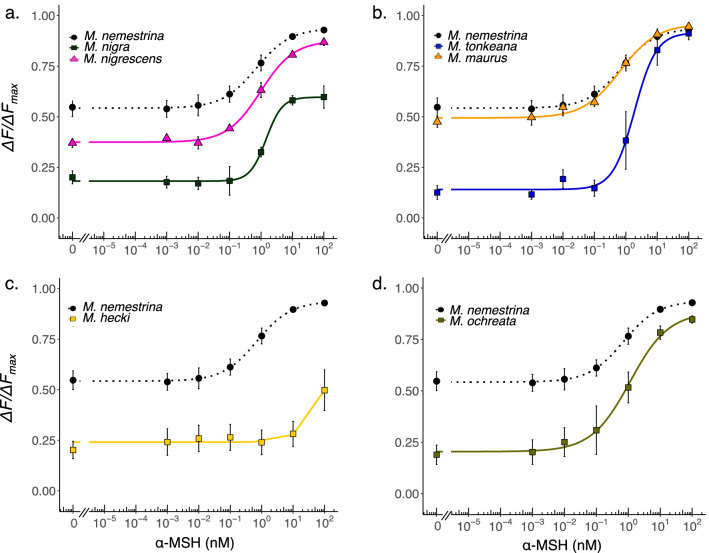
Dose–response curve for MC1R of Sulawesi macaques and *M. nemestrina*. The response of (**a**) *M. nemestrina, M. nigra* and *M. nigrescens)*, (**b**) *M. tonkeana* and *M. maurus,* (**c**) *M. hecki*, (**d**) *M. ochreata*. Each point represents the mean ± standard error of the mean (SEM) determined from at least three independent measurements.

Intracellular cAMP production under various concentrations of α-MSH is presented in Fig. 5. The responses of MC1R to agonist α-MSH differed substantially among species. α-MSH dose-dependently activated MC1R of all species, except for *M. hecki* MC1R. *M. nigra* MC1R showed a significantly lower maximal cAMP production than those for MC1R of other species (pairwise *t*-test, P < 0.05, BH-adjusted; Table 2). The EC_50_ values were similar for *M. maurus* (0.663 ± 0.339 nM) and *M. nemestrina* (0.709 ± 0.363 nM). The EC_50_ values for MC1R of *M. nigrescens* (0.968 ± 0.490 nM), *M. ochreata* (1.125 ± 0.477 nM), *M. nigra* (1.458 ± 0.803 nM) and *M. tonkeana* (1.886 ± 0.583 nM) were slightly higher than those of *M. nemestrina* MC1R (Table 2). Exceptionally, *M. hecki* MC1R showed cAMP accumulation with 100 nM α-MSH stimulation. Because saturation was not reached, we could not determine the EC_50_ values (Fig. 5c).

To determine the key residues affecting MC1R function, we designed several key mutants of MC1R based on SIFT and PROVEAN analyses. To understand the functional changes in both northern and southern clusters, we constructed vectors of the predicted ancestral northern (G304E mutant of *M. nigra*) and ancestral southern sequences (S104G mutant of *M. tonkeana*) and measured cAMP accumulation with/without α-MSH stimulation (Table 2). Both mutants, *M. nigra*_G304E (Supplementary Fig. S2a) and *M. tonkeana_*S104G (Supplementary Fig. S2b), showed higher constitutive activation than that of each wild-type protein and had similar sensitivity to α-MSH to that of *M. nemestrina* (EC_50_ = 0.485 ± 0.145 nM and 0.696 ± 0.304 nM, respectively). To evaluate the effects of key amino acids on MC1R function, we also tested the Y267C mutant of *M. hecki* MC1R (Supplementary Fig. S2c). As expected, *M. hecki*_Y267C MC1R (EC_50_ = 0.316 ± 0.180 nM) rescued the binding affinity to α-MSH, with significantly higher cAMP production and a left shift of the dose–response curve compared with those of wild-type *M. hecki* MC1R. Different from the above three mutants, the P153H mutant of *M. maurus* (EC_50_ = 1.164 ± 0.306 nM; Supplementary Fig. S2d) had significantly reduced basal cAMP production and a right shift of the dose–response curve compared with those of wild-type *M. maurus*. These results indicate that species-specific MC1R variants in the six species independently affect either basal activity or agonist-induced responses.

## Discussion

We observed that the amino acid sequences of MC1R are conserved within each species (n = 10) and identified fixed species-specific amino acid substitutions in six closely related *Macaca* species. We further analyzed the functional features of MC1R itself and in response to the agonist α-MSH by a cAMP assay. We found that all six species-specific MC1R variants exhibited divergent basal activity and agonist-induced cAMP performance compared with those of the predicted ancestral sequences of the northern and southern clusters and *M. nemestrina* MC1R. And we identified the key residues responsible for MC1R function by site-directed mutagenesis.

We observed low nucleotide diversity in *MC1R* in each species of Sulawesi macaques, with an average of 0.067 × 10^–2^, which was similar to the estimate for human *MC1R* in African populations (π = 0.07 × 10^−2^)^21^ and three times lower than *MC1R* diversity in both *M. nemestrina* (π = 0.21 × 10^−2^) and *M. fascicularis* (π = 0.20 × 10^−2^; Supplemental Table S5). Our results suggested that the low *MC1R* diversity of *M. nigra* and *M. nigrescens* was a consequence of purifying selection for a dark coat color. Purifying selection on *MC1R* appears to be common in non-human primates^22^. Moreover, Nakayama and colleagues compared the nucleotide sequences of *MC1R* in 18 *Macaca* species and concluded that the gene was under purifying selection in the ancestral lineage of macaques and the *silenus* group^15^. In the present study, we verified that the amino acid sequence of *MC1R* is highly conserved in each Sulawesi macaque based on large population sizes and obtained additional functional information for each haplotype.

Numerous non-synonymous substitutions in *MC1R* have been identified in mammals. In humans, many loss-of-function variants are associated with red hair color^23^. Notably, the R151C, R160W, and D294H mutations in MC1R are strongly associated with pale skin and red hair in Eurasians. Opposite to the dark coat color in Sulawesi macaques, we found that basal activity levels of MC1R were dramatically diminished in *M. hecki*, *M. ochreata*, *M. nigra,* and *M. tonkeana*, resulting from C267Y, R306C, E304G, and G104S mutations, respectively. Exceptionally, *M. maurus* has the most ancestral coat color (brownish)^16^. Consistent with this observation, *M. maurus* MC1R displayed comparable basal activity and agonist-induced activity to those of *M. nemestrina* MC1R. The increase of constitutive activity in *M. maurus* can be explained by a change in the secondary structure by the H153P substitution in IL2. Accordingly, the five species-specific mutations (G104S, H153P, C267Y, E304G, and R306C) in Sulawesi macaques identified in the present study independently affected the basal activity of MC1R.

The most influential substitution was C267Y (*M. hecki*) with respect to agonist binding activity. C267 and C275 form disulfide bonds between TM6 and TM7 affecting the tertiary structure of the receptor^24^. Single point mutation of C267 to glycine results in a complete loss of NDP-MSH binding. However, the serine mutant retained some agonist binding ability, though weaker than that of the wild-type locus^25^. Similarly, we found that C267Y exhibited agonist-independent basal activity and a cAMP response to α-MSH with an extremely high threshold (> 100 nM). In humans, the plasma concentration of α-MSH is 21.30 ± 0.63 nM^26^. Our results suggested that the natural C267 mutant with the hydrophobic residue tyrosine in *M. hecki* retained low agonist-induced activation; however, activity might be constant under the low concentration of α-MSH in the body.

MC1R EL1 is small compared with most GPCRs and deletions of EL1 resides are associated with melanism in the gray squirrel^27^, jaguar, and jaguarundi^28^. In the present study, the G104S variant (*M. tonkeana*) exhibited decreased basal activity and a slight right-shift in agonist-induced activity. This variant has also been detected in gibbons^22^ and buffaloes^29^. Miao et al. speculated that the black coat color is associated with the allele carrying the G104S substitution^29^, but do they cause melanism is unclear. In silico functional prediction suggested that the G104S substitution is deleterious in buffaloes, and this was further supported by our functional results for the G104S variant in *M. tonkeana*. Our result suggests that G104S substitution could decrease MC1R activity and hence it is not likely to be causative for the melanism.

It is not clear how these species-specific substitutions in MC1R became fixed in Sulawesi macaques. MC1R acts as a genetic switch that determines whether dark eumelanin or light pheomelanin is produced for the regulation of coat color. Loss-of-function mutations usually lead to lighter, yellowish color, including in humans. In the present study, we found that most species-specific mutations resulted in reducing MC1R activity in vitro. Based on *M. nemestrina* as the extant ancestor, the predicted ancestral haplotypes of southern and northern clusters showed significantly reduced basal activity, with further reductions occurring independently in *M. nigra*, *M. tonkeana*, *M. hecki*, and *M. ochreata* (Supplementary Fig. S1). The basal activity and agonist-induced activity evolved independently in each *Macaca* species on Sulawesi Island, consistent with results for lemurs and platyrrhines^14^. Haitina et al. suggested catarrhine primates displayed conservation of dose-dependent α-MSH binding and activation, with variation in basal activity. However, we found that C267Y in *M. hecki* almost led to loss of α-MSH-induced cAMP production. The changes in MC1R function caused by these novel mutations are not simply limited to melanism in coat color in Sulawesi macaques, consistent with the results of Haitina et al.^14^ and Nakayama et al.^15^. For example, we did not investigate the promoter region of *MC1R* or the sequences and expression levels of other pigmentation-related genes (e.g., *POMC* (proopiomelanocortin) and *TYR* (Tyrosinase)). Increased levels of α-MSH might lead to increased melanin production in the island population^30^. The limit of in vitro heterologous expression system using HEK293 cells might be one of the causes for the discrepancy between the protein molecular properties and pigmentation phenotypes in the monkeys. There might be a systematic difference in transfection efficiency, protein localization, dimerization, and internalization in vitro assay system with transient transfection. Further explorations of the expression levels and regulatory regions of *MC1R* and other pigmentation-related genes in vivo are needed to explain coat color divergence in the Sulawesi macaques.

Sulawesi macaques differ in patterns of unmelanized or light part on the forearm, cheek, upper arm, thighs and hind-shanks. In rhesus macaques, Bradley et al. did not find significant differences in the patterns of gene expression in comparing dark, intermediate and light hair^31^. Their results suggested the coat color variation from light to dark in rhesus macaques was unlikely to be due to differences in expression levels of key pigmentation genes; *MITF* (Melanocyte Inducing Transcription Factor)*, MC1R*, *MGRN1* (Mahogunin Ring Finger 1)*, ATRN* (Attractin)*, SLC24A5* (Solute Carrier Family 24)*, TYRP1*(Tyrosinase-related Protein 1) and *DCT* (Dopachrome-tautomerase)^31^. Hence, the expression of MC1R might be not causative to fine tuning of pattern difference in the case for Sulawesi macaques. *ASIP* gene plays a key developmental role in color patterning. Spatio-temporal regulation of ASIP can further modify MC1R activity. Mundy and Kelly suggested that mutations in *ASIP* coding region were not involved in color changes among closely related primate species^32^. Allele-specific expression of ASIP in body part has been found to be responsible for color pattern differences in mice^33^. We speculate that expression and regulatory differences at ASIP might play an important role in pattern variation in Sulawesi macaques. A protein expression analysis would further improve our understanding of variation among species and body parts.

Sulawesi macaques are morphologically differentiated, though individuals with intermediate traits, presumably hybrids, have been reported in the border zone of each species’ distribution^34–37^. Gene flow between Sulawesi macaque species has presumed from the intermediate morphological characteristics^38^. The possibility of gene flow in a hybrid zone is also supported by an analysis of microsatellite markers^39^. Previous genetic analyses of hemoglobin^18^ and *TAS2R38*^40^ identified shared common haplotypes among Sulawesi macaques; however, we found that *MC1R* shows species-specific variants, without shared haplotypes. Divergence in *MC1R* was the greatest between *M. maurus* and *M. nigra*, suggesting a high correlation with geographical distance. While the relationship between the protein sequence and coat color phenotype is still unclear, *MC1R* could be a species-specific marker gene for Sulawesi macaques. In addition to the apparent change-of-function MC1R variants for cAMP production in Sulawesi macaques, MC1R may have other important functions that are unique to each species.

## Methods

### Study subjects

Six endemic species in the genus *Macaca* on Sulawesi Island, Indonesia were evaluated, *M. tonkeana*, *M. hecki*, *M. nigrescens*, *M. nigra*, *M. maurus*, and *M. ochreata.* Although some studies have suggested that *M. ochreata* can be separated into two species, *M. ochreata* and *M. brunnescens*, we treat these species as a subspecies of *M. ochreata* according to the IUCN Red List^41^. Saliva samples were obtained from 51 captive animals (Supplementary Table S1) collected for a previous study^40^. The saliva was scrubbed with cotton swabs and samples were stored in 2 mL tubes with 1 mL of lysis buffer consisting of 0.5% sodium dodecyl sulfate, 100 mM ethylenediaminetetraacetic acid, 100 mM Tris–HCl, and 10 mM NaCl at room temperature^42^. This collection method was approved by the Animal Ethics Commission of the Research and Community Service Institute, Ministry of Research, Technology and Higher Education, Bogor Agricultural University (Permission number no. 35-2016 IPB).

### Genotyping of *MC1R*

*MC1R* orthologues in *M. mulatta*, *M. nemestrina*, and *M. fascicularis* were sequenced for comparison. Genomic DNAs of Sulawesi macaques and an individual of *M. mulatta* were extracted from buccal swabs of monkeys using the QIAamp DNA Investigator Kit (Qiagen GmbH, Hilden, Germany). Genomic DNAs of *M. nemestrina* (N = 10) and *M. fascicularis* (N = 7) were extracted from blood samples using the DNeasy Blood and Tissue Kit (Qiagen)^40^.

To amplify and sequence the entire coding region of the *MC1R* gene, primers (MC1R-F: 5′ ATGAGCTAAGCAGGACACC 3′; MC1R-R: 5′ CAACACCTTCAGAGGTCAGT 3′) were designed using the Primer3Plus website (website: http://www.bioinformatics.nl/cgi-bin/primer3plus/primer3plus.cgi). *MC1R* was amplified using ExTaq DNA Polymerase (Takara Bio Inc., Shiga, Japan) by PCR under the following conditions: initial denaturation at 94 °C for 10 min, 45 cycles of denaturation at 94 °C for 10 s, annealing at 56 °C for 30 s, and extension at 72 °C for 1 min, followed by a final extension at 72 °C for 10 min. PCR products were sequenced using BigDye Terminator v. 3.1 (Applied Biosystems, Carlsbad, CA) and the sequencing products were separated by capillary electrophoresis using a 3130*xl* Genetic Analyzer (Applied Biosystems).

### Reconstruction of *MC1R* haplotypes and selection analysis

Sequences of intact MC1R of all the samples were aligned using MEGA X. A maximum-likelihood (ML) of the six species was reconstructed with 1000 bootstrap replicates. The ancestral amino acid sequences of each clusters were inferred using ML-based ancestral reconstruction in MEGA X. Multisite haplotypes were reconstructed from sequence data using DnaSP v. 6.12.03^43^. The genealogical relationships among haplotypes were constructed, rooted with the *MC1R* sequence of *Papio hamadryas* (Accession number AY205105.1), using the median-joining algorithm implemented in PopART^44^. To test for a signal of selection in the partial *MC1R* gene or lineage, we used the CODEML program in PAML 4^45^. The ratio ω (d N/d S) is a measure of selective pressure, where ω = 1, ω < 1 and ω > 1 correspond to neutral evolution, purifying and positive selection, respectively. Three models, branch, site and branch-site models were used to analyze the selection of *MC1R* in Sulawesi macaques. The phylogenetic tree was referred to RADseq data in Evans et al.^19^. Firstly, we used site models (M0: null, M1: nearly neutral selection, M2: positive selection, M3: discrete, M7: beta, and M8: beta and ω > 1) to determine candidates of positively selected sites. Model fit was evaluated using likelihood ratio tests (LRT). Subsequently, to test whether the ω ratio was different among lineages, the melanism lineage (*M. nigra* and *M. nigrescens*), the other four species (*M. hecki*, *M. tonkeana*, *M. maurus*, *M. ochreata*) and the reference branch (*M. nemestrina* and *M. mulatta*) were set up and tested by branch model. Additionally, branch-site model was tested to determine whether positive selection occurred on a few sites in a small number of lineages. The Bayes empirical Bayes (BEB) analysis was applied to calculate posterior probabilities of positively selected sites.

### Expression vector construction and site-directed mutagenesis

The entire coding region of *MC1R* was amplified from genomic DNA of *M. hecki*, *M. nigra*, *M. tonkeana*, *M. maurus*, and *M. ochreata* and the *MC1R* gene fragment tagged with a 27 bp Flag-tag at the N-terminal end was inserted into the pcDNA3.3 mammalian expression vector using the pcDNA™3.3-TOPO™ TA Cloning™ Kit (Invitrogen, Carlsbad, CA). The insertion and orientation of the fragment were confirmed by direct sequencing. The vectors for *M. nigrescens* and *M. nemestrina* A (38V) were generated from the wild-type *M. nigra* sequence by site-directed mutagenesis using the QuikChange Lightning Site-Directed Mutagenesis Kit (Agilent Technologies, Santa Clara, CA). To identify the key amino acid substitutions, we predicted the functional effect of single amino acid substitutions using the SIFT and PROVEAN algorithms in the PROVEAN web server^46^. The site-directed mutations in pcDNA3.3 vectors were also generated using the QuikChange Lightning Site-Directed Mutagenesis Kit.

### Cell culture

Human embryonic kidney 293T (HEK293T) cells were provided by Dr. Matsunami (Duke University) via Dr. Misaka (The University of Tokyo) for the functional analysis. Cells were cultivated in a 5% CO_2_ incubator at 37 °C with Dulbecco’s modified Eagle’s medium (Fujifilm Wako Pure Chemical Corporation, Osaka, Japan) supplemented with 10% fetal bovine serum (Thermo Fisher Scientific).

### cAMP assay using cultured cells

A cAMP assay was used to evaluate the constitutive activity and α-MSH agonist-induced activity of MC1R variants. In vitro experiments were performed in accordance with the guidelines of Kyoto University. The protocol was approved by the Genetic Research Committee of the Agency for Health, Safety and Environment, Kyoto University to H.I. (no. 151031 and 200101). The constructed MC1R vector was transiently transfected into HEK293T cells using Lipofectamine 2000 (Life Technologies, Inc., Carlsbad, CA). The *M. nemestrina* (38V) MC1R expression vector was adopted as a positive control and the empty pcDNA3.3 vector was defined as the mock control. Transfections were performed in 96-well plates when cells reached 70–80% confluence. Transfection mixtures were prepared using 1 ng of MC1R vector, 0.3 μL of Lipofectamine 2000, and 10 μL of Opti-MEM per well. The transfection mixtures were preincubated for 20 min at room temperature before their addition to plates. Then, the plates were incubated at 37 °C under 5% CO_2_ for 24 h. The cAMP assays were performed using the cAMP-Gs Dynamic Kit (Cisbio, Codolet, France). Briefly, on the day of the cAMP assay, α-MSH was diluted with Stimulation Buffer 1 to obtain a final concentration range of 10^−12^ to 10^−7^ M. Then, 7500 cells were added to each well of a white-walled 384-well plate and stimulated with different concentrations of α-MSH (Sigma-Aldrich, St. Louis, MO) for 30 min at room temperature. The cells were also stimulated with 20 µM forskolin (final concentration 10 µM; Cisbio) to normalize for cell numbers^47^. Following the protocol for the cAMP Dynamic Kit, fluorescence signals at 665 and 620 nm were detected using the FlexStation 3 Microplate Reader (Molecular Devices Japan, Inc., Tokyo, Japan). Values are expressed as Δ*F* (using ratios of 665 nm/620 nm for the assay and 665 nm/620 nm for the mock). Data are expressed as Δ*F/*Δ*F*_*max*_^48^, which is the ratio of the ligand-dependent increase (Δ*F)* to the maximal production of cAMP under activation by 20 µM forskolin (Δ*F*_*max*_). Δ*F/*Δ*F*_*max*_ values were fitted by the function *f(x)* = *min* + (*max* − *min*)/(1 + (*x*/EC_50_)^h^)), where *x* is the ligand concentration and *h* is the Hill coefficient, using the drc package in R^49^. At least three independent measurements were conducted for each vector. Data are reported as mean values ± standard error of the mean (SEM).

## Supplementary Information

Supplementary Tables.

Supplementary Figure S1.

Supplementary Figure S2.

## Data Availability

DNA sequences are available in DDBJ; DDBJ accessions LC632229 to LC632296.
