# Supplementary material for: Functional divergence of the pigmentation gene melanocortin-1 receptor (MC1R) in six endemic Macaca species on Sulawesi Island
Source: Sci Rep. 2022 May 9;12:7593. doi: 10.1038/s41598-022-11681-z (PMC9085793; doi:10.1038/s41598-022-11681-z)
Supplement: Supplementary file 2 — Supplementary Figure S1. [file 41598_2022_11681_MOESM2_ESM.docx]

Supplementary Figure S1. Phylogeny tree referred to autosomal RADseq data generated by Evans et al., 2017. The ancestral amino acid at site 38 was predited based on the maximum likelihood analysis. Up arrow indicated that amino acid substitution in red led to drastic increase of basal activity in MC1R, while down arrow indicated that amino acid substition in red caused decrease of basal activity in MC1R. The branch model analysis indicated that the melanism lineage (*M. nigra* and *M. nigrescens*) has significantly lower ω (ω = 0.08565) than the other four species (ω = 0.96785) and the outgroup (ω = 0.8560). From Riley (2010) with permission, illustration courtesy of Stephen Nash.

**REFERENCE**

Evans, B. J. *et al.* Speciation over the edge: Gene flow among non-human primate species across a formidable biogeographic barrier. *R. Soc. Open Sci.* **4**, 170351 (2017).

Riley, E. P. The endemic seven: Four decades of research on the Sulawesi macaques. *Evolutionary Anthropology. Issues, News, and Reviews* **19**, 22-36 (2010).
