# Supplementary material for: Functional divergence of the pigmentation gene melanocortin-1 receptor (MC1R) in six endemic Macaca species on Sulawesi Island
Source: Sci Rep. 2022 May 9;12:7593. doi: 10.1038/s41598-022-11681-z (PMC9085793; doi:10.1038/s41598-022-11681-z)
Supplement: Supplementary file 3 — Supplementary Figure S2. [file 41598_2022_11681_MOESM3_ESM.docx]

Supplementary Figure S2. Dose–response curve for MC1R of Sulawesi macaques and its mutants*.* The response of (a) *M. nigra* and the G304E mutant of *M. nigra* (consensus of the northern branch), (b) *M. tonkeana* and its mutant S104G *(*consensus of the southern branch*),* (c) *M. maura* and its mutant *P153H,* (d) *M. hecki* and its mutant Y267C. Each point represents the mean ± standard error of the mean (SEM) determined from at least three independent measurements.
